# Superoxide Dismutase 3 Controls the Activation and Differentiation of CD4^+^T Cells

**DOI:** 10.3389/fimmu.2021.628117

**Published:** 2021-02-25

**Authors:** Gaurav Agrahari, Shyam Kishor Sah, Chul Hwan Bang, Yeong Ho Kim, Tae-Yoon Kim

**Affiliations:** ^1^ Laboratory of Dermato-Immunology, College of Medicine, The Catholic University of Korea, Seoul, South Korea; ^2^ Department of Reconstructive Sciences, Center for Regenerative Medicine and Skeletal Development, UConn Health, Farmington, CT, United States

**Keywords:** superoxide dismutase 3, CD4^+^T cells, activation, proliferation, signaling pathways

## Abstract

Superoxide dismutase 3 (SOD3), a well-known antioxidant has been shown to possess immunomodulatory properties through inhibition of T cell differentiation. However, the underlying inhibitory mechanism of SOD3 on T cell differentiation is not well understood. In this study, we investigated the effect of SOD3 on anti-CD3/CD28- or phorbol myristate acetate (PMA) and ionomycin (ION)-mediated activation of mouse naive CD4^+^ T cells. Our data showed that SOD3 suppressed the expression of activation-induced surface receptor proteins such as CD25, and CD69, and cytokines production. Similarly, SOD3 was found to reduce CD4^+^T cells proliferation and suppress the activation of downstream pathways such as ERK, p38, and NF-κB. Moreover, naïve CD4^+^T cells isolated from global SOD3 knock-out mice showed higher expression of CD25, CD69, and CD71, IL-2 production, proliferation, and downstream signals compared to wild-type CD4^+^T cells. Whereas, the use of DETCA, a known inhibitor of SOD3 activity, found to nullify the inhibitory effect of SOD3 on CD4^+^T cell activation of both SOD3 KO and wild-type mice. Furthermore, the expression of surface receptor proteins, IL-2 production, and downstream signals were also reduced in Th2 and Th17 differentiated cells upon SOD3 treatment. Overall, our data showed that SOD3 can attenuate CD4^+^T cell activation through modulation of the downstream signalings and restrict CD4^+^T cell differentiation. Therefore, SOD3 can be a promising therapeutic for T cell-mediated disorders.

## Introduction

Superoxide dismutase 3 (SOD3) is one of the isoforms of superoxide dismutase enzyme that scavenges the superoxide radicals, and are predominant in the extracellular matrix and on cell surfaces with a smaller fraction in the plasma and extracellular fluids (1, 2). Along with well-established antioxidant and anti-inflammatory properties, SOD3 is also known for its immunomodulatory functions. SOD3 suppressed the release of pro-inflammatory cytokines from hyaluronic acid fragments (HAFs)-stimulated bone marrow-derived macrophages and dendritic cells (DCs). In addition, SOD3 also found to attenuate DCs maturation through downregulation of cell surface expression of major histocompability complex II (MHC II), CD80, and CD86 without altering CD44 or Toll-like receptor 4 expressions (3, 4). However, this inhibition of DCs maturation was found to be SOD3 specific and was not affected by either SOD1 or SOD2 (4). Studies also showed that SOD3 modulates CD4^+^T cell regulation and differentiation in both *in vivo* and *in vitro* conditions. SOD3 found to inhibit CD4^+^T cell activation, proliferation, and differentiation when co-cultured with activated DCs and under differentiation conditions (4). Similarly, SOD3 knock-out CD4^+^ T cells were found to be more differentiated into Th17 cells compared to T cells isolated from wild-type mice (5). However, the inhibitory mechanism of SOD3 on the regulation of CD4^+^T cell function is not well studied.

CD4^+^T helper cells play a major role in regulating acquired immune response through promoting intracellular killing by macrophages, antibody production by B cells, and clonal expansion of cytotoxic T cells (6). However, aberrant activation and proliferation of T cells contribute to several autoimmune diseases such as rheumatoid arthritis (7, 8), multiple sclerosis (9) and psoriasis (10). Upon activation, T cells produce variety of cytokines such as interleukin-2, tumor necrosis factor-α, and interferon-γ, which further alleviates its proliferation and immune-regulatory functions and also acts on other immune cells to regulate their functions (11). Similarly, the activation and expression of cytokines in T cells are regulated by a number of signaling cascades including the nuclear factor-κB (NF- κB) and mitogen-activated protein kinase (MAPK) pathways (11, 12). After activation of CD4^+^ T cells, it simultaneously differentiates into various subsets of T helper cells depending upon the polarizing microenvironment (13). Thus, activation which is the initial event in T cell differentiation can be targeted to prevent the further differentiation of T cells.

In this study, we hypothesized that SOD3 can control the activation of naïve CD4+ T cells and thus it can suppress the differentiation of T cells. The effect of SOD3 on the expression of surface activation receptors, cytokines production, proliferation and downstream signals in activated and differentiated T cells were investigated. Our results illustrate the possible role of SOD3 in controlling the activation and differentiation of T cells.

## Materials and Methods

### CD4^+^ T Cell Isolation, Culture, Treatment, and Activation

Mouse naïve CD4^+^ T cells were isolated from spleen and lymph nodes of 4–6 weeks old C57BL6 wild-type and whole body SOD3 knock out mice as previously described (14). Briefly, sub-populations of CD4^+^ T cells were obtained from single cell suspension of spleen and lymph nodes by using mouse CD4^+^ T cell enrichment column (MCD4C-1000, R&D Systems). The cells were then subjected to fluorescence-activated cell sorting (FACS) using fluorescence conjugated antibodies against CD4 (550954, BD Bioscience), CD44 (11-0441-82, eBioscience), CD62L (12-0621-82, eBioscience) and CD25 (17-0259-42, eBioscience). Naïve CD4+ T cells were sorted as CD4^+^CD44^-^CD62L^+^CD25^-^ populations (**Supplementary Figure 1A**). Prior to activation, 2×10^6^ naïve CD4^+^T cells/mL were seeded on 6 well-plates and pre-treated with recombinant human SOD3 (rh SOD3; 100 U/mL, 200 U/mL, and 300 U/mL) for 1 h in Roswell Park Memorial Institute (RPMI)-1640 medium (CA059-050; Gendepot, Houston, Tx) supplemented with 10% fetal bovine serum, 100 U/mL penicillin, and 100 μg/mL streptomycin (CA 005-010; Gendepot) at 37°C in an incubator. After pre-treatment of SOD3, naïve CD4^+^T cells were then activated with either combination of 3 μg/mL of mouse anti-CD3 (Catalog No. MAB484-100, R&D systems) and 3 μg/mL of anti-CD28 (Catalog No. 16-0281-85, eBioscience) or with 100 ng/mL of phorbol myristate acetate (PMA) (Catalog No.16561-29-8, Sigma-Aldrich) and 300 ng/mL of ionomycin (ION) (Catalog No.13909, Sigma-Aldrich) for 24 h.

### Cell Surface Receptor Analysis

APC-conjugated anti-mouse CD25 and PE-conjugated anti-mouse CD69 were purchased from Biolegends. Isolated naïve CD4+T cells were pre-treated with rh SOD3 (200 U/mL) for 1 h. After pre-treatment of SOD3, naïve CD4^+^T cells were then activated with combinations of 3 μg/mL of mouse anti-CD3 and 3 μg/mL of anti-CD28, or with 100 ng/mL PMA and 300 ng/mL ION for 24 h. Cells were then harvested, washed and stained with APC-conjugated anti-mouse CD25 and PE-conjugated anti-mouse CD69. Cell surface expression of CD25 and CD69 were analyzed by flow cytometry.

### T Cell Differentiation

Isolated naïve CD4+ T cells were seeded at a concentration of 2×10^6^ cells/mL in 6 well-plates and pre-treated with 200 U/mL of SOD3 for 1 h. The cells were then treated with differentiating cytokines (for Th2 differentiation: 10 ng/mL rh mouse IL-4 (MIL 4-25, JW CreaGene Inc.), 10 ng/mL rh mouse IL-2 (CYT-370, Prospec), 10 µg/mL mouse anti-IFN-γ (130-095-729, Miltenyi Biotec); for Th17 differentiation: 20 ng/mL rh mouse IL-6 (14-8061-62, eBioscience), 2 ng/mL rh humanTGF-β (14-8348-62, eBioscience), 10 µg/mL mouse anti-IL-4 (554434, BD Pharmingen), and 10 µg/mL mouse anti-IFN-γ (130-095-729, Miltenyi Biotec) along with 3 μg/mL of mouse anti-CD3(MAB484-100, R&D systems) and 3 μg/mL of CD28 (16-0281-85, eBioscience).

### Enzyme Linked Immunosorbent Assay (ELISA)

Isolated naïve CD4+ T cells were seeded at a concentration of 2×10^6^ cells/mL in 6 well-plates and pre-treated with 100 U/mL, 200 U/mL, and 300 U/mL of SOD3 for 1 h. The cells were then treated with either anti-CD3 (3 μg/mL) and anti-CD28 (3 μg/mL) or PMA (100 ng/mL) and ION (300 ng/mL) for 24 h. The levels of cytokines released were measured from cell-free supernatant by using mouse ELISA kits. IL-2 (554424, 554426), IFN-γ (554410, 551216) ELISA reagents were purchased from BD Pharmingen. IL-4 (431104), IL-5 (431204), IL-6 (431304), IL-10 (431414) and TNF-α (430904) ELISA kits were purchased from Biolegend. IL-13 (M1300CB) ELISA kit was purchased from R&D Systems. ELISA was performed according to manufacturer’s instructions.

### cDNA Synthesis and Quantitative Real-Time PCR (qRT-PCR) Analysis

Total RNA was isolated from cells by using TRIzol reagent (Catalog No. 15596018, Life Technologies, Invitrogen). Complementary DNA was synthesized from 1 μg of total RNA using the PrimeScript™ RT reagent Kit (RR047A, Clontech Takara Bio INC, Japan) and qRT-PCR was performed using LightCycler 96 (Roche Diagnostics, Mannheim, Germany). Glyceraldehyde 3-phosphate dehydrogenase (GAPDH) was used as an endogenous control. The used primers and their sequences can be found in supplementary sections (**Supplementary Table 1**).

### Cell Proliferation Assay

For Proliferation measurement, isolated naïve T cells were plated in 96-well plates at 2×10^5^ cells/mL in 100 μL per well of culture media. Cells were then pre-treated with SOD3 for 1 h followed by activation with either anti-mouse CD3 and CD28 or PMA and ionomycin for further 3 days. Cell proliferation was measured with BrdU cell proliferation assay kit (Catalog No. 2750, Millipore) following the manufacturer’s instruction.

### Western Blot Analysis

Cells were lysed in ice-cold radioimmunoprecipitation(RIPA) lysis buffer (Catalog No. 89901, Thermo Scientific, Rockford, USA) containing protease inhibitor cocktail (11873580001, Roche Diagnostic, Germany) and phosphatase inhibitor cocktail (P5726, Sigma-Aldrich). Protein concentrations were determined by BCA protein assay kit (Catalog No. 23225, Thermo Scientific, Rockford, USA) as described by manufacturer instructions. Equal amounts of proteins were separated by sodiumdodecyl sulfate-polyacrylamide gel electrophoresis (SDS-PAGE) and transferred to polyvinylidene difluoride membrane (PVDF). After blocking, membranes were incubated with primary antibodies such as p-p38 (9211S, Cell Signaling), p38 (9212S, Cell Signaling), p-ERK1/2 (9101S, Cell Signaling), ERK1/2 (9102S, Cell Signaling), p-NF-κBp65 (SC-136548, Santa Cruz), NF-κBp65 (SC-109, Santa Cruz) and GAPDH (SC-32233, Santa Cruz) at 1:1,000 dilutions for overnight at 4°C. Membranes were then washed and incubated with horseradish-peroxidase conjugated secondary antibodies (1:5,000 dilutions) for 2 h at room temperature. The blots were then detected with western blot detection kit (WesternBrightTMECL, USA).

### ROS Measurement

For the determination of ROS production during T cell activation and differentiation, isolated T cells were first pre-treated with 10 µM of dihydroethidium (DHE) (D1168, Invitrogen) and dichlorofluorescein diacetate (DCFDA) (ab113851, abcam) and incubated in dark at 37°C for 30 min. The cells were then treated with SOD3 (200 U/mL), mouse anti-CD3 and anti-CD28, and differentiating cytokines for Th2 and Th17 cells, and incubated for 10, 30, and 60 min. Fluorescent were measured at 500 nm; 600 nm for DHE and 485 nm; 525 nm for DCFDA.

### SOD3 Purification and Activity Assay

SOD3 was purified as previously described (15). Briefly, SOD3 plasmids were transfected into human embryonic kidney cells (HEK293E) and media were collected after every 48 h. The collected media were filtered and loaded on HiTrap Chelating HP column (GE Healthcare).The recombinant SOD3 was verified by western blot with SOD3 antibody as previous described (16). SOD3 activity in the culture medium was analyzed by SOD assay kit as per manufacturer’s instruction (S311, Dojindo Molecular Technologies, Japan).

### Statistical Analysis

Statistical differences were analyzed by one-way analysis of variance (ANOVA) followed by Tukey test. All results represent three independent experiments. P <0.05 was considered statistically significant (*p < 0.05, **p < 0.01, ***p < 0.001).

## Results

### SOD3 Suppressed the Expression Levels of Surface Activation Markers in CD4^+^T Cells

**Figure 1:**
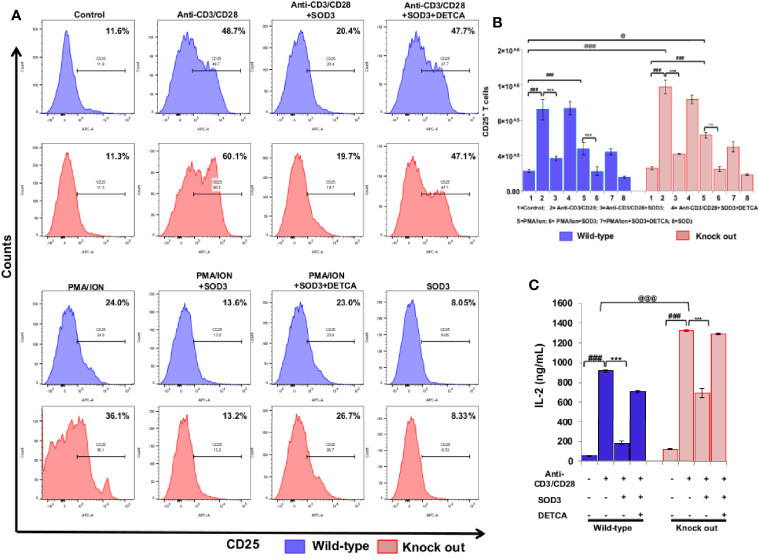
Effect of SOD3 on surface activation markers. **(A)** CD4^+^T cells isolated from wild type and SOD3 knock out mice were first pre-treated with 200 U/mL of SOD3 and DETCA (10 µM) for 1 h and then treated with anti-CD3/CD28 (3 μg/mL) or PMA (100 ng/mL) and Ionomycin (300 ng/ml) for 24 h. Expression levels of surface proteins were determined by FACS. **(B)** Absolute cell numbers of CD25 positive T cells. **(C)** IL-2 productions were determined from cell free supernatant by ELISA. All experiments were performed in triplicate. The data shown represent one of three independent experiments. Data are expressed as mean ± standard deviation. ^#^p<0.05, ^##^p<0.01, ^###^p<0.001 (Control group vs. anti-CD3/CD28 or PMA/ION-treated group); ^*^p<0.05, ^**^p<0.01, ^***^p<0.001 (anti-CD3/CD28 or PMA/ION-treated group vs. SOD3 and anti-CD3/CD28 or PMA/ION-treated group); ^@^p<0.05, ^@@^p<0.01, ^@@@^p<0.001 (wild-type vs SOD3 Knock out).

**Figure 2:**
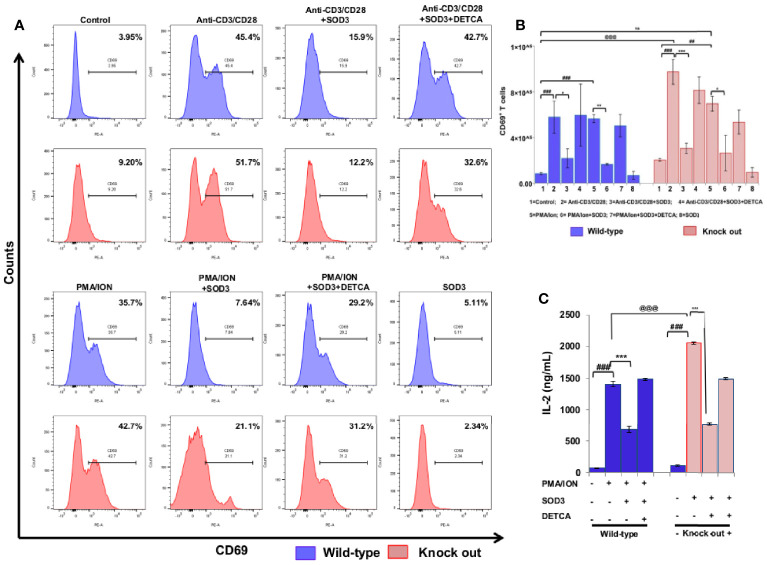
SOD3 suppressed CD69 expression and IL-2 production. CD4^+^T cells were isolated from SOD3 knock out and wild type mice first pre-treated with 200 U/mL of SOD3 and DETCA (10 µM) for 1 h and then treated with anti-CD3/CD28 (3 μg/mL) or PMA (100 ng/mL) and Ionomycin (300 ng/ml) for 24 h. **(A)** Expression levels of surface proteins were analyzed by FACS. **(B)** Absolute cell numbers of CD69 positive T cells. **(C)** IL-2 productions were determined from cell free supernatant by ELISA. All experiments were performed in triplicate. The data shown represent one of three independent experiments. Data are expressed as mean ± standard deviation. ^#^p<0.05, ^##^p<0.01, ^###^p<0.001 (Control group vs. anti-CD3/CD28 or PMA/ION-treated group); ^*^p<0.05, ^**^p<0.01, ^***^p<0.001 (anti-CD3/CD28 or PMA/ION-treated group vs. SOD3 and anti-CD3/CD28 or PMA/ION-treated group); ^@^p<0.05, ^@@^p<0.01, ^@@@^p<0.001 (wild-type vs SOD3 Knock out).

Activation of naïve CD4^+^ T cells results into the expression of various molecules on their surface which clearly distinguished them from naïve CD4^+^T cells (17) and the activation of naïve CD4^+^T cell is measured by increased expression of CD25, CD69, and CD71 (18–20). Thus, we first aimed to examine the dose-dependent effect of SOD3 on the expression levels of surface receptor proteins such as CD25, CD69, and CD71 at mRNA level. Here, we found that SOD3 significantly suppressed both anti-CD3/CD28 and PMA/ION-induced expression levels of CD25, CD69 and CD71 in CD4^+^T cells. However, there was no significant difference in inhibitory effect between different doses of SOD3 (**Supplementary Figure 2A**). To further evaluate the inhibitory effect of SOD3 on CD4^+^T cell activation, we isolated the naïve CD4^+^T cells from whole body SOD3 knock-out (KO) mice and also used an inhibitor of SOD3 activity, DETCA. Here, we found that the expression levels of activation markers such as CD25 and CD69, and IL-2 production were more upregulated in T cells isolated from SOD3 KO mice compared to wild-type mice under both anti-CD3/CD28 and PMA/ION-induced activation at protein level. Whereas, the use of DETCA constrain the inhibitory effect of SOD3 on CD4^+^T cell activation. In addition, SOD3 suppressed the expression of CD25, CD69, and IL-2 production in both wild-type and KO mice (**Figures 1A–C** and **2A–C**). Similar results were also observed at transcript level (**Supplementary Figures 2** and **3**).

### Effect of SOD3 on the Cytokines Produced by Activated-T Cells

Activated-T cells are also known to secrete various cytokines that function to modulate both innate and adaptive immunity (19, 20). Thus, we next evaluated the effect of SOD3 on the cytokine secretion in activated-T cells. Our data showed that the expression levels of cytokines such as IL-2, IL-4, IL-5, IL-6, IL-10, IL-13, IFN-γ, and TNF-α were found to be reduced in SOD3 treated groups compared to anti-CD3/CD28, and PMA/ION-activated groups at both mRNA and protein levels (**Tables 1** and **2**).

**Table 1:** Effects of SOD3 on cytokine production.

S. No.	Cytokines	Stimulation	Mean relative mRNA expression levels/GAPDH				
			Control	Positive	+ SOD3		
					100 U/ml	200 U/ml	300 U/ml
**1**	**IL-2**	Anti-CD3+CD28	1	+ 4.18 ± 0.02**^###^**	−1.85 ± 0.27^***^	−2.64 ± 0.11^***^	− 2.99 ± 0.09^***^
		PMA+ION		+7.88 ± 0.60**^###^**	−1.54 ± 0.17^**^	−2.54 ± 0.15^**^	−5.00 ± 0.01^***^
**2**	**IL-4**	Anti-CD3+CD28	1	+36.81 ± 1.85**^###^**	−36.19 ± 0.49^***^	−21.36 ± 0.16^***^	−36.44 ± 0.06^***^
		PMA+ION		+108.45 ± 11.78**^###^**	−26.11 ± 4.08^**^	−78.28 ± 4.42^**^	−84.65 ± 1.94^***^
**3**	**IL-5**	Anti-CD3+CD28	1	+ 44.57 ± 7.56**^###^**	−40.53 ± 0.20^***^	−45.54 ± 0.15^***^	−45.53 ± 0.02^***^
		PMA+ION		+101.18 ± 0.50**^###^**	−41.26 ± 9.52^**^	−85.61 ± 0.65^***^	−92.70 ± 1.02^***^
**4**	**IL-6**	Anti-CD3+CD28	1	+ 63.50 ± 0.52**^###^**	−34.44 ± 0.23^***^	−55.84 ± 0.13^***^	−57.55 ± 0.11^***^
		PMA+ION		+419.24 ± 6.18**^###^**	−38.77 ± 13.09^**^	−170.34 ± 6.12^***^	−177.67 ± 19.00^***^
**5**	**IL-10**	Anti-CD3+CD28	1	+ 28.71 ± 2.62**^###^**	−13.02 ± 2.77^**^	−21.73 ± 0.35^**^	−27.95 ± 0.93^***^
		PMA+ION		+578.58 ± 90.53**^##^**	−575.90 ± 0.05^***^	−575.26 ± 0.96^***^	−575.71 ± 1.32^***^
**6**	**IL-13**	Anti-CD3+CD28	1	+17.70 ± 2.19**^###^**	−9.39 ± 0.99^**^	−13.07 ± 0.95^**^	−18.20 ± 0.21^***^
		PMA+ION		+139.38 ± 50.47**^###^**	−139.28 ± 0.07^**^	−139.661 ± 0.12^**^	−139.43 ± 0.15^**^
**7**	**TNF-α**	Anti-CD3+CD28	1	+ 8.35 ± 0.13**^###^**	−2.91 ± 0.40^***^	−4.32 ± 0.09^***^	−5.15 ± 0.12^***^
		PMA+ION		+49.73 ± 0.24**^###^**	−14.97 ± 1.40^***^	−26.47 ± 0.95^***^	−39.05 ± 0.74^***^
**8**	**IFN-γ**	Anti-CD3+CD28	1	+158.39 ± 10.14**^###^**	−125.56 ± 0.99^***^	−146.38 ± 0.89^***^	−149.74 ± 0.37^***^
		PMA+ION		+82.86 ± 0.82**^###^**	−27.56 ± 0.85^***^	−49.31 ± 1.69^***^	−65.61 ± 2.56^***^

CD4^+^T cells were first pre-treated with 100, 200, and 300 U/mL of SOD3 for 1 h and then treated with anti-CD3/CD28 (3 μg/mL) or PMA (100 ng/mL) and Ionomycin (300 ng/ml) for 24 h. Expression levels of cytokines were determined by qRT-PCR. All experiments were performed in triplicate. Data are expressed as mean ± standard deviation. ^#^p < 0.05, ^##^p < 0.01, ^###^p < 0.001 (Control group vs. anti-CD3/CD28 or PMA/ION-treated group); ^*^p < 0.05, ^**^p < 0.01, ^***^p < 0.001 (anti-CD3/CD28 or PMA/ION-treated group vs. SOD3 and anti-CD3/CD28 or PMA/ION-treated group).

**Table 2:** SOD3 suppressed the release of cytokines in activated T cells.

S. No.	Cytokines	Stimulation	Average cytokine level (pg/mL)				
			Control	Positive	+ SOD3		
					100 U/ml	200 U/ml	300 U/ml
**1**	**IL-2**	**Anti-CD3+CD28**	29.93 ± 0.89	55.53 ± 3.16**^##^**	40.11 ± 1.68^**^	34.24 ± 0.27^**^	31.64 ± 1.72^**^
		**PMA+Ionomycin**		349.67 ± 0.75**^###^**	341.62 ± 0.88^**^	337.95 ± 0.95^**^	312.40 ± 1.37^***^
**2**	**IL-4**	**Anti-CD3+CD28**	29.40 ± 1.03	235.75 ± 6.38**^###^**	157.69 ± 3.24^**^	138.19 ± 8.92^***^	73.97 ± 7.30^***^
		**PMA+Ionomycin**		249.72 ± 33.65**^###^**	155.45 ± 12.89^*^	131.89 ± 11.35^**^	108.38 ± 0.81^**^
**3**	**IL-5**	**Anti-CD3+CD28**	43.86 ± 1.35	70.25 ± 0.55**^###^**	43.47 ± 1.10^***^	36.87 ± 1.65^***^	31.44 ± 0.55^***^
		**PMA+Ionomycin**		89.26 ± 5.49**^##^**	36.48 ± 4.39^***^	32.60 ± 1.10^***^	29.50 ± 2.20^***^
**4**	**IL-6**	**Anti-CD3+CD28**	51.59 ± 1.65	186.58 ± 1.65**^###^**	166.41 ± 6.03^*^	153.61 ± 8.78^**^	95.03 ± 2.74^***^
		**PMA+Ionomycin**		330.10 ± 22.49**^###^**	284.72 ± 1.10^*^	60.12 ± 3.84^***^	52.75 ± 3.29^***^
**5**	**IL-10**	**Anti-CD3+CD28**	584.15 ± 1.25	773.75 ± 26.69**^##^**	651.08 ± 13.34^*^	582.63 ± 3.39^**^	527.80 ± 3.44^***^
		**PMA+Ionomycin**		1,179.49 ± 40.03**^###^**	905.35 ± 0.71^**^	1,000.21 ± 53.38^**^	830.36 ± 26.69^***^
**6**	**IL-13**	**Anti-CD3+CD28**	104.36 ± 1.07	142.00 ± 3.01**^###^**	116.43 ± 3.01^**^	112.88 ± 8.03^***^	112.53 ± 1.50^**^
		**PMA+Ionomycin**		136.31 ± 8.03**^##^**	132.77 ± 5.02^*^	128.15 ± 0.50^*^	127.97 ± 1.26^*^
**7**	**TNF-α**	**Anti-CD3+CD28**	28.21 ± 3.15	49.36 ± 2.62**^##^**	39.71 ± 2.62^*^	39.34 ± 1.07^*^	43.43 ± 1.57^*^
		**PMA+Ionomycin**		229.75 ± 34.12**^##^**	189.66 ± 23.62^*^	159.23 ± 36.22^*^	132.87 ± 16.80^*^
**8**	**IFN-γ**	**Anti-CD3+CD28**	4025.79 ± 3.12	4,101.93 ± 14.05**^###^**	3,844.81 ± 37.46^*^	3,706.86 ± 92.08^*^	3,670.44 ± 74.91^**^
		**PMA+Ionomycin**		4,374.51 ± 46.82**^##^**	3,758.73 ± 37.46^**^	3,606.44 ± 81.15^**^	3,447.52 ± 84.28^**^

CD4^+^T cells were first pre-treated with 100, 200, and 300 U/mL of SOD3 for 1 h and then treated with anti-CD3/CD28 (3 μg/mL) or PMA (100 ng/mL) and Ionomycin (300 ng/ml) for 24 h. Expression levels of cytokine productions were determined by ELISA. All experiments were performed in triplicate. Data are expressed as mean ± standard deviation. ^#^p < 0.05, ^##^p < 0.01, ^###^p < 0.001 (Control group vs. anti-CD3/CD28 or PMA/ION-treated group); ^*^p < 0.05, ^**^p < 0.01, ^***^p < 0.001 (anti-CD3/CD28 or PMA/ION-treated group vs. SOD3 and anti-CD3/CD28 or PMA/ION-treated group).

### SOD3-Mediated Suppression of CD4^+^ T Cell Activation

To further evaluate the inhibitory effect of SOD3 on CD4^+^T cell activation, we isolated the naïve CD4^+^T cells from whole body SOD3 knock-out (KO) mice and also used an inhibitor of SOD3 activity, DETCA. Here, we found that the expression levels of activation markers and IL-2 production were more upregulated in T cells isolated from SOD3 KO mice compared to wild-type mice under both anti-CD3/CD28 and PMA/ION-induced activation. Whereas, the use of DETCA constrain the inhibitory effect of SOD3 on CD4^+^T cell activation (**Figures 2A, B**).

### SOD3 Altered T Cell Proliferation

**Figure 3:**
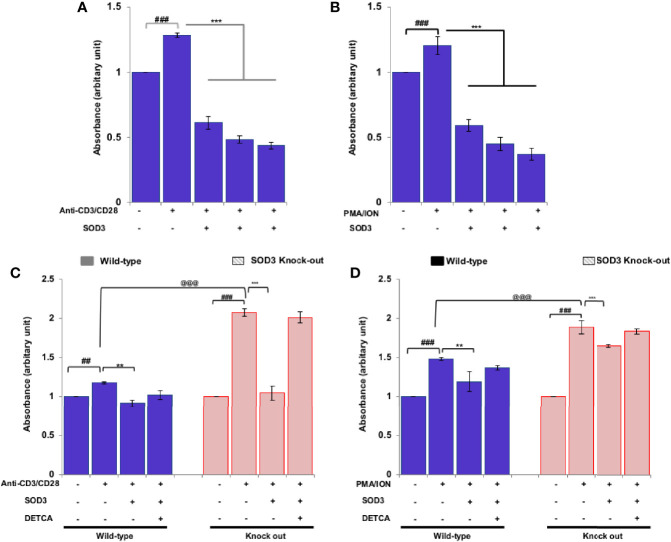
Effects of SOD3 on CD4^+^T cell proliferation. CD4^+^T cells were first pre-treated with 100, 200, and 300 U/mL of SOD3 for 1 h and then treated with **(A)** anti-CD3/CD28 (3 μg/mL) or **(B)** PMA (100 ng/mL) and Ionomycin (300 ng/ml) for 3 days. CD4^+^T cells isolated from wild type and SOD3 knock out mice were first pre-treated with 200 U/mL of SOD3 and DETCA (10 µM) for 1 h and then treated with **(C)** anti-CD3/CD28 (3 μg/mL) or **(D)** PMA (100 ng/mL) and Ionomycin (300 ng/ml) for 3 days. Cell proliferation was measured with BrdU cell proliferation assay kit. All experiments were performed in triplicate. Data are expressed as mean ± standard deviation. ^#^p<0.05, ^##^p<0.01, ^###^p<0.001 (Control group vs. anti-CD3/CD28 or PMA/ION-treated group); ^*^p<0.05, ^**^p<0.01, ^***^p<0.001 (anti-CD3/CD28 or PMA/ION-treated group vs. SOD3 and anti-CD3/CD28 or PMA/ION-treated group); ^@^p<0.05, ^@@^p<0.01, ^@@@^p<0.001 (wild-type vs SOD3 knock out).

Activation of naïve CD4^+^T cells enables the cell to enter cell cycle phase and undergoes rapid proliferation (17). Thus, to determine the consequences of SOD3 on the activation-induced proliferation of naïve CD4^+^T cells, we measured the proliferative response by BrdU uptake after 3 days of incubation. Our data showed that SOD3 suppressed both anti-CD3/CD28 and PMA/ION-induced proliferation of activated T cells (**Figures 3A, B**). Interestingly, T cells isolated from SOD3 KO mice were found to be more proliferated than wild-type mice, and treatment with SOD3 reduced the proliferation in both wild-type and SOD3 KO-isolated CD4^+^T cells. Similarly, the use of DETCA inhibits the suppressive effect of SOD3 on the activation-induced proliferation of T cells isolated from both wild-type and SOD3 KO mice (**Figures 3C, D**).

### SOD3 Suppress the Activation of Downstream p38/ERK, and NF-κB Signaling Pathways

**Figure 4:**
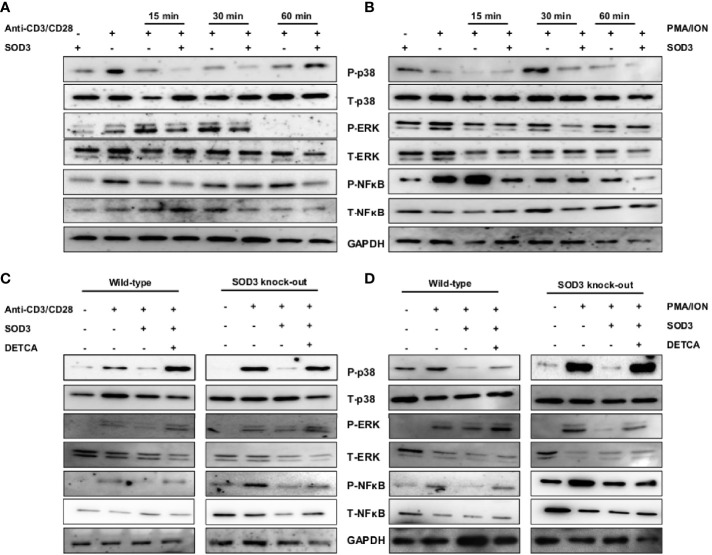
SOD3 suppress the activation-induced downstream signaling pathways. CD4^+^T cells were first pre-treated with 200 U/mL of SOD3 for 1 h and then treated with **(A)** anti-CD3/CD28 (3 μg/mL) or **(B)** PMA (100 ng/mL) and Ionomycin (300 ng/ml) for indicated time periods. CD4^+^T cells isolated from wild type and SOD3 knock out mice were first pre-treated with 200 U/mL of SOD3 and DETCA (10 µM) for 1 h and then treated with **(C)** anti-CD3/CD28 (3 μg/mL) or **(D)** PMA (100 ng/mL) and Ionomycin (300 ng/ml) for 30 min. Activation of p38, ERK1/2 and NF-κB were analyzed by western blot. All experiments were performed in triplicate. Band densities of blot are shown in **Supplementary Figures 5A–D**.

Crosslinking of anti-CD3/CD28 and the use of PMA/ION results into the activation of various downstream signaling pathways (21, 22). Thus, we next determined to evaluate the effect of SOD3 on anti-CD3/CD28 and PMA/ION-induced activation of downstream MAPK and NF-κB signaling pathways. Here, we found that SOD3 suppressed the activation of p38, ERK, and NF-κB signals under both anti-CD3/CD28, and PMA/ION-mediated activation of naïve CD4^+^T cells (**Figures 4A, B**). In addition, the activation of these signals was also found to be suppressed with SOD3 treatment in naïve CD4^+^T cells isolated from SOD3 KO mice. Similarly, the use of DETCA suppressed the inhibitory effect of SOD3 on the activation of these signaling pathways (**Figures 4C, D**).

### SOD3 Reduced the Expression of Surface Markers, IL-2 Production, Proliferation, ROS, and Downstream Signals in Th2 and Th17 Cells

**Figure 5:**
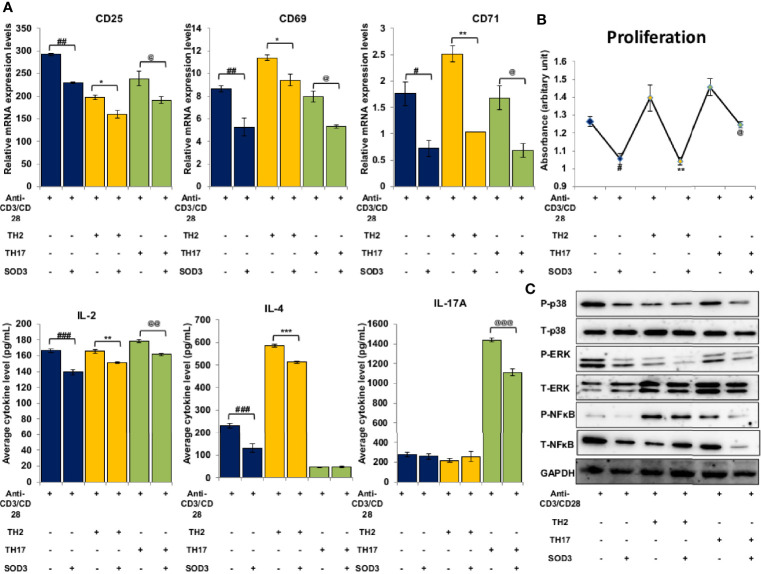
SOD3 suppressed the activation markers, proliferation and signaling pathways in Th2 and Th17 differentiated cells. CD4^+^T cells were first pre-treated with 200 U/mL of SOD3 for 1 h and then treated with anti-CD3/CD28 (3 μg/mL) and differentiating cytokines. **(A)** Expression levels of surface proteins and cytokine productions were analyzed by qRT-PCR after 24 h of incubation. **(B)** Proliferation and viability were determined by Brdu assay and PrestoBlue cell viability reagent respectively on day 3. **(C)** Activation of signaling pathways were analyzed by western blot on day 3. All experiments were performed in triplicate. Data are expressed as mean ± standard deviation. ^#^p<0.05, ^##^p<0.01, ^###^p<0.001 (anti-CD3/CD28 vs. SOD3 and anti-CD3/CD28); ^*^p<0.05, ^**^p<0.01, ^***^p<0.001 (Th2vs. SOD3 and Th2); ^@^p<0.05, ^@@^p<0.01, ^@@@^p<0.001 (Th17 vs. SOD3 and Th17). Band densities of western blot are shown in **Supplementary Figure 5E**.

The activation of T cells is followed by the differentiation of T cell lineage under the influence of polarizing cytokines. Thus, we next aimed to determine the effect of SOD3 on T cell activation markers under Th2 and Th17 differentiation conditions. Here, we found that with SOD3 treatment, the expression levels of surface activation markers such as CD25, CD69, and CD71, and IL-2 production were reduced in activated, Th2, and Th17 differentiated cells (**Figure 5A**). Similarly, the proliferation of differentiated cells was also found to be diminished upon SOD3 treatment (**Figure 5B**). We next determined the effect of SOD3 on the superoxide anion and H_2_O_2_ levels in activated and differentiated cells. The levels of both superoxide anions and H_2_O_2_ were found to be reduced in SOD3-treated groups. However, the reduction in H_2_O_2_ levels were not statistically significant in SOD3-treated groups (**Supplementary Figure 4A, B**). Moreover, the activation of downstream p38, ERK, and NF-κB signals were also found to be diminished in SOD3-treated groups (**Figure 5C**).

## Discussion

In this study, we utilized two different conditions to investigate the effect of SOD3 on T cell activation. Crosslinking of anti-CD3 and anti-CD28 antibodies mimic receptor aggregation and activates the T cells similar to physiological conditions mediated by T cell and antigen-presenting cell interactions. On the other hand, the mitogen phorbol myristate acetate (PMA) is structurally similar to diacylglycerol (DAG) and known to activate downstream NF-κB signaling pathways whereas mitogen ionomycin (ION) induces calcium influx for the activation of T cells. Thus, in this study, we aimed to determine the effect of SOD3 under both receptor-dependent and receptor-independent activation of CD4^+^ T cells.

Activated T cells are known to express various receptor proteins such as CD25 (the IL-2 receptor), CD69 (the early activation antigen), and CD71 (the transferrin receptor) on their cell surface which clearly differentiated them from naïve CD4^+^T cells (17, 23). Thus, to study the effect of SOD3 on T cell activation, we first examined the expression levels of the receptor proteins. Here, we found that SOD3 significantly reduced the expression levels of surface activation markers such as CD25, CD69, and CD71. The expression of these surface receptors are tightly regulated through MAPK signaling cascades (24, 25). Our data indicates that the inhibitory effect of SOD3 on the expression level of surface receptors may be mediated through controlled regulation of the downstream signaling cascades such as p38, and ERK. In addition, the higher expression of these receptors in CD4^+^T cells isolated from whole body SOD3 KO mice further emphasized the importance of SOD3 in controlling the expression of these receptors during activation.

T cell is known to produce wide variety of cytokines upon activation that acts both in autocrine and paracrine manner to affect the proliferation and functions of their own and regulates the function of other immune cells (26, 27). Consistent with the previous study (4), we also found that SOD3 can significantly downregulate the production of IL-2 in T cells. Besides IL-2, further analysis also demonstrated that SOD3 inhibited the production of IL-4, IL-5, IL-6, IL-10, IL-13, TNF-α, and IFN-γ in both anti-CD3/CD28 and PMA/ION-induced activation of naïve CD4^+^T cells. Along with surface receptor, the transcription of the cytokine genes in activated T cells is also regulated by the various downstream signaling pathways such as NF-κB and MAPK (28, 29). Here, we showed that SOD3 can downregulate the activation of NF-κB, p38, and ERK signals in both anti-CD3/CD28 and PMA/ION-induced activation of T cells. Thus, through restricting the expression of these cytokine productions in T cells, SOD3 may suppress T cells-mediated immune response and thereby reduce inflammatory response.

A study by Kwon et al. showed that SOD3 can significantly downregulate the proliferation of Th17 cells (4), whereas a study by Lee et al. showed that T cells isolated from SOD3 KO mice showed higher proliferation than wild-type mice under Th17 differentiation condition (5). Previously, SOD3 found to downregulate the cell cycle gene such as cdk2 and cdk4 in mesenchymal stem cells under serum-starved conditions (30) and suppressed UVB-induced proliferation in melan-a cells (31). Thus, in this study, we examined the effect of SOD3 in activation-induced CD4^+^T cells proliferation and found that SOD3 can suppress the proliferation of CD4^+^T cells. Activation of T cells mediated by cross-linking of TCR and CD28 enables the cell to transit from the G_0_ to the G_1_ phase of the cell cycle (32, 33). However, the antigen receptor stimulation alone is not sufficient to promote the cell cycle and thus require additional cytokine stimulation to continue through the cell cycle and progress to the S phase (34, 35). Cytokine IL-2 is well known to provide an additional signal which leads to the activation of cyclin-dependent kinases protein and promotes the cell to enter the S phase of the cell cycle (34). In addition, ROS generation during T cell activation stimulates the generation of IL-2 and other pro-proliferative genes (36). The suppression of IL-2 and its receptor (CD25) on SOD3 treatment suggest that the inhibitory effect of SOD3 on T cell proliferation induced by both anti-CD3/CD28 and PMA/ION is not due to non-specific toxicity of SOD3 but may be due to inhibition of growth-promoting cytokines, and ROS production. Thus, we believe that SOD3 can inhibit the cell to enter the cell cycle phase and suppress the proliferation of cells without affecting its viability.

**Figure 6:**
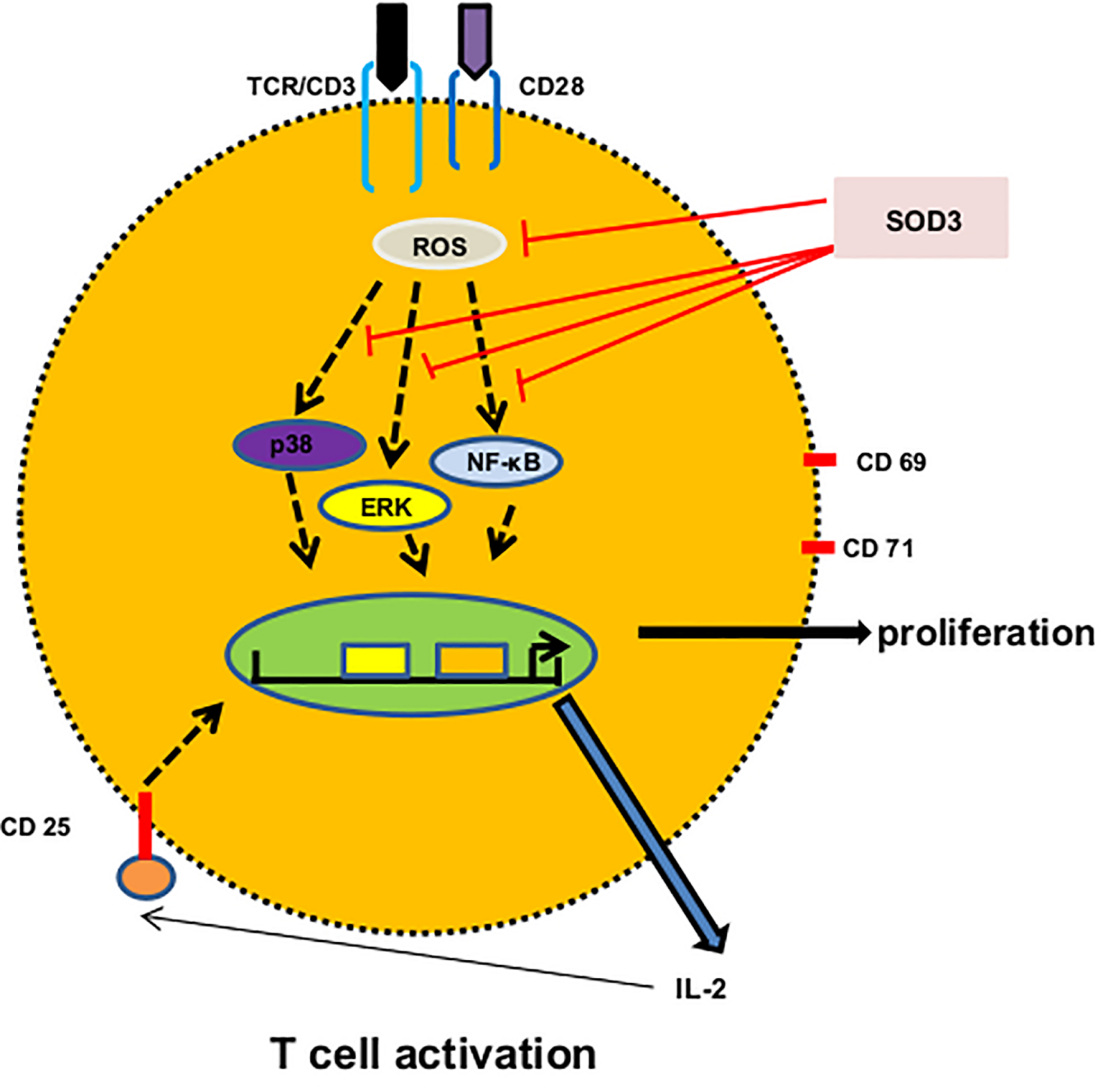
A proposed mechanism of SOD3 on CD4^+^T cell activation. Engagement of anti-CD3 and anti-CD28 with their respective receptors trigger ROS production and activation of p38, ERK and NF-κB signaling which result into the expression of surface receptor proteins, cytokines and proliferation. SOD3 suppressed these events through scavenging ROS production and regulating subsequent signaling.

Previously, we have shown that SOD3 can control the cathelicidin-mediated activation of p38 and ERK1/2 signals in mast cells (37). Similarly, SOD3 is found to inhibit the hyaluronic acid fragments-mediated NF-κ B recruitment to the promoters of target genes in macrophages (3). In addition, SOD3 is also found to inhibit the activation of p38, ERK1/2 and NF-κB signaling pathways both in *in vitro* and *in vivo* under various inflammatory conditions in both enzymatically and non-enzymatic manners (3, 37, 38). Even though SOD3 is secreted into the extracellular matrix, studies showed that the heparin binding domain of SOD3 acts as nuclear localization signal for secretion, re-uptake and nuclear translocation (39, 40). Ligation of anti-CD3 and anti-CD28 with their respective receptors on T cells are known to activate various signaling events particularly NF-κB, and MAPK, and these signaling pathways activate NF-κ B and AP-1 transcription factors (22, 41). The transactivation of these transcription factors result into the regulation of several genes responsible for expression of intracellular and cell surface proteins, and secretion of cytokines (17, 22). The levels of ROS are known to increase during T cell activation and these ROS appear to correlate with the phosphorylation kinetics and plays an important role in activation of downstream signaling pathways by inactivating protein tyrosine phosphatases (PTPs) and thus, by enhancing the activation of protein tyrosine kinases (42). The activation of MAPK and NF-κB has been shown to rely upon ROS produced during T cell activation (43, 44). Therefore, SOD3-mediated downregulation of surface activation proteins and cytokines productions may be mediated through control activation of these signaling pathways and SOD3 may limits the activation of these signaling cascades, at least in part, through modulation of ROS production (**Figure 6**).

SOD3 is also well known to exhibit anti-tumor activity. Overexpression of SOD3 inhibited breast carcinoma cell growth and invasion through reduced expression of heparanase (45). Similarly, Overexpression of SOD3 acts as a tumor suppressor in PC-3 prostate cancer (46). The anti-tumor effect of SOD3 is mainly regulated through its antioxidative function. In addition, SOD3 negatively regulates pro-oncogenic signaling pathways and also provoke DNA damage-induced apoptosis (47). Along with anti-tumor activity, SOD3 also exhibit anti-autoimmune potential. Overexpression of SOD3 found to ameliorate several auto-immune diseases such as arthritis, psoriasis (48) and the alternation in the level of SOD3 found to be associated in inflammatory bowel diseases and systemic lupus erythematosus (49, 50). These studies emphasized the importance of SOD3 as an anti-tumor and anti-autoimmune bio-compound.

Once activated, CD4^+^T cell differentiate into specific lineage depending upon the polarizing microenvironment. Previously, SOD3 showed to inhibit the differentiation of Th2 and Th17 cells (4). However, the inhibitory mechanisms of SOD3 on T cell differentiation were remained elusive. Here, we showed that SOD3 may inhibit the early activation step of CD4^+^T cells, thereby inhibits its differentiation into Th2 and Th17 cells. Our data showed that the activation markers, IL-2 productions, proliferation, and activation-induced signaling pathways such as p38, ERK1/2, and NF-κB were decreased in both Th2 and Th17 cells. Activation of p38, ERK, and NF- κB found to regulate the differentiation of Th2 and Th17 cells (51–56). Thus, we believe that SOD3 may modulate the activation of these signaling pathways and restrict the differentiation of CD4^+^T cells into Th2 and Th17 cells.

Upon TCR stimulation, T cells are known to produce superoxide anion and H_2_O_2_ within few minutes and play an important role in the regulation of signaling events during T cell activation (36, 43, 44). Our study showed that SOD3 suppress the ROS production in T cells. Studies showed that treatment with various antioxidants inhibited the proliferation and IL-2 production in T cells (57). Similarly, activation of T cells is also known to increase the extracellular production of SOD1 (58). In addition, manganese SOD found to reduce the activation-induced mitochondrial ROS production, and NF-κB and AP-1 transcription (59). These studies emphasized the important role of ROS and antioxidants in the regulation of T cell activation. Therefore, SOD3, being an antioxidant can regulate the activation of T cells.

SOD3 found to exhibit its function in both enzymatic and non-enzymatic manners. Mass spectrometry analysis showed that SOD3 interacts with receptors, including EGF and TNF receptors; adaptors; adhesion molecules; kinases; phosphatases; apoptosis-related factors; and nicotinamide adenine dinucleotide phosphate (NADPH) oxidase (4). In addition, we also found that SOD3 can interact with IL-4R and histamine 4 receptor (60). TCR ligation-induced ROS play a role in regulating signaling events such as MAPK and NF-κB during T cell activation. Here, we showed that SOD3 suppressed the activation-induced ROS production in T cells. Thus, we believe that the inhibitory mechanism of SOD3 may be mediated through both enzymatic and non-enzymatic manners.

Despite the importance of CD4^+^T cells in regulating adaptive immunity during host defense against microorganisms, inappropriate and hyperactivation of T cells can contribute to various inflammatory diseases (7–10). Thus, it is very important to control the abnormal activation of these cells to overcome the deleterious consequences. SOD3, being a natural antioxidant with no harmful effect reported yet, can be therapeutically used for managing CD4^+^T cell-mediated inflammatory diseases. However, a detailed study should be conducted for the safe and effective use of SOD3 as an alternative therapy in T cell-mediated disorders.

## Data Availability Statement

The original contributions presented in the study are included in the article/**Supplementary Material**. Further inquiries can be directed to the corresponding author.

## Ethics Statement

The animal study was reviewed and approved by The Catholic Ethics Committee of Catholic University of Korea.

## Author Contributions

GA contributed to the design, conceptualization, formal analysis, investigation, methodology, visualization, and writing. SS, CB, and YK contributed to the investigation, methodology, and writing. T-YK contributed to conceptualization, supervision, and validation. All authors contributed to the article and approved the submitted version.

## Funding

This research was supported by the Bio and Medical Technology Development Program of the National Research Foundation (NRF) funded by the Korean government (MSIT) (no. 2016M3A9B6903020).

## Conflict of Interest

The authors declare that the research was conducted in the absence of any commercial or financial relationships that could be construed as a potential conflict of interest.

## Abbreviations

SOD3, superoxide dismutase 3; PMA, phorbol myristate acetate; ION, ionomycin; Th cells, T helper cells; ERK, Extracellular signal-regulated kinases; NF-κB, Nuclear factor-κB; DETCA, diethyldithiocarbamate trihydrate.
