# Supplementary material for: Superoxide Dismutase 3 Controls the Activation and Differentiation of CD4+T Cells
Source: Front Immunol. 2021 Feb 25;12:628117. doi: 10.3389/fimmu.2021.628117 (PMC7947887; doi:10.3389/fimmu.2021.628117)
Supplement: Supplementary file 1 [file DataSheet_1.pdf]

Supplementary Figure S1: Sorting strategy for the isolation of mouse naïve CD4$^+$T cells. (A) Lymphocytes are gated by size. (B, C) singlet cells are discriminated from doublet cells. (D) CD4$^+$CD25$^+$ cells are natural Treg cells whereas CD4$^+$CD25$^-$ cell populations are non-T reg cells. (E) Naïve CD4$^+$CD44$^-$CD62L$^+$ were sorted against CD4$^+$CD44$^+$CD62L$^-$ (memory T cells).

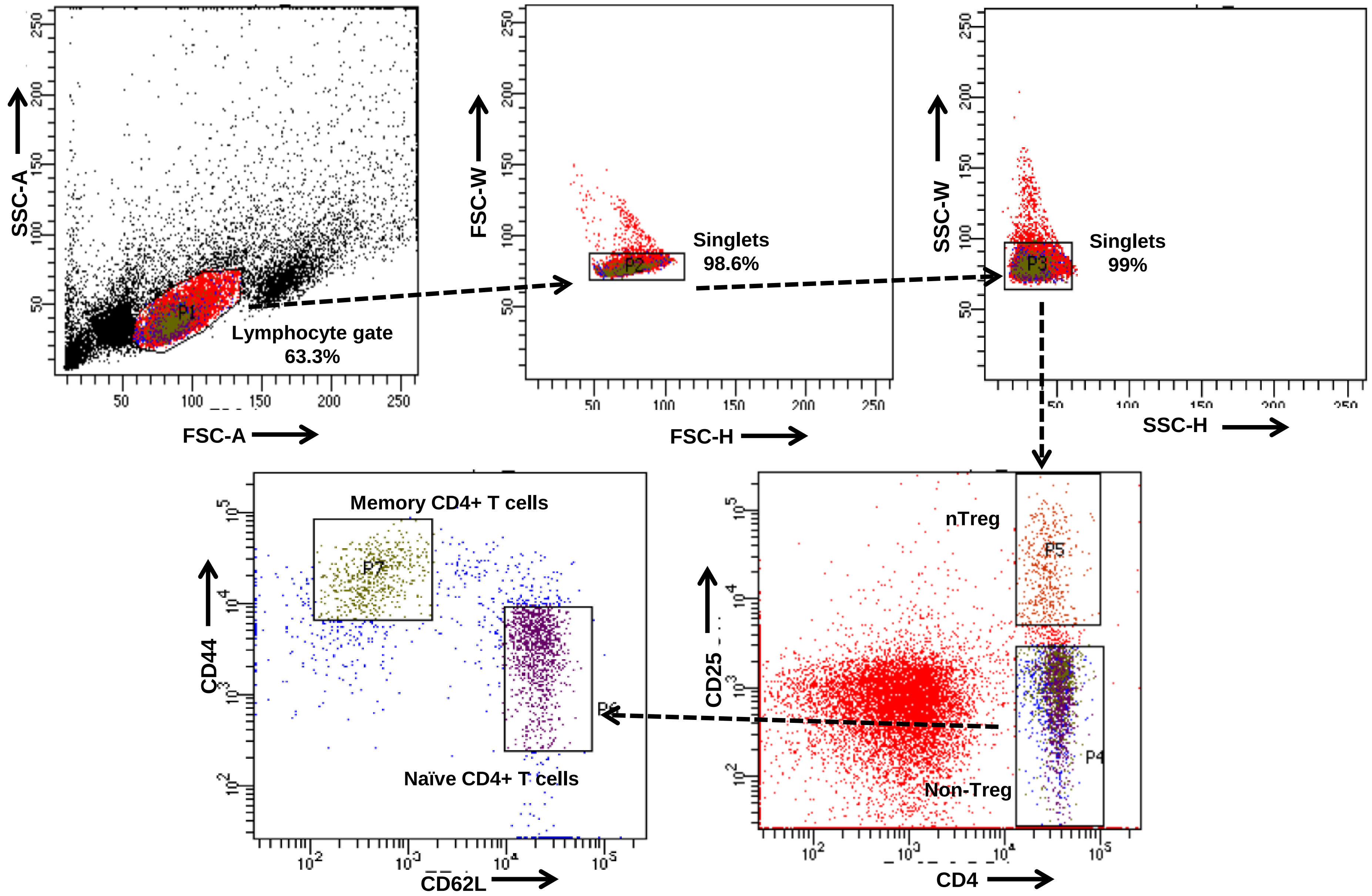

Supplementary Figure S2: Effect of SOD3 on surface activation markers. $CD4^+$T cells were first pre-treated with 100, 200 and 300 U/mL of SOD3 for 1 hour and then treated with (a) anti-CD3/CD28 (3 μg/mL) or (b) PMA (100 ng/mL) and Ionomycin (300 ng/ml) for 24 hours. Expression levels of surface proteins were determined by qRT-PCR. All experiments were performed in triplicate. Data are expressed as mean ± standard deviation. $^{\#}p<0.05$, $^{\#\#}p<0.01$, $^{\#\#\#}p<0.001$ (Control group vs. anti-CD3/CD28 or PMA/ION-treated group); $^{*}p<0.05$, $^{**}p<0.01$, $^{***}p<0.001$ (anti-CD3/CD28 or PMA/ION-treated group vs. SOD3 and anti-CD3/CD28 or PMA/ION-treated group).

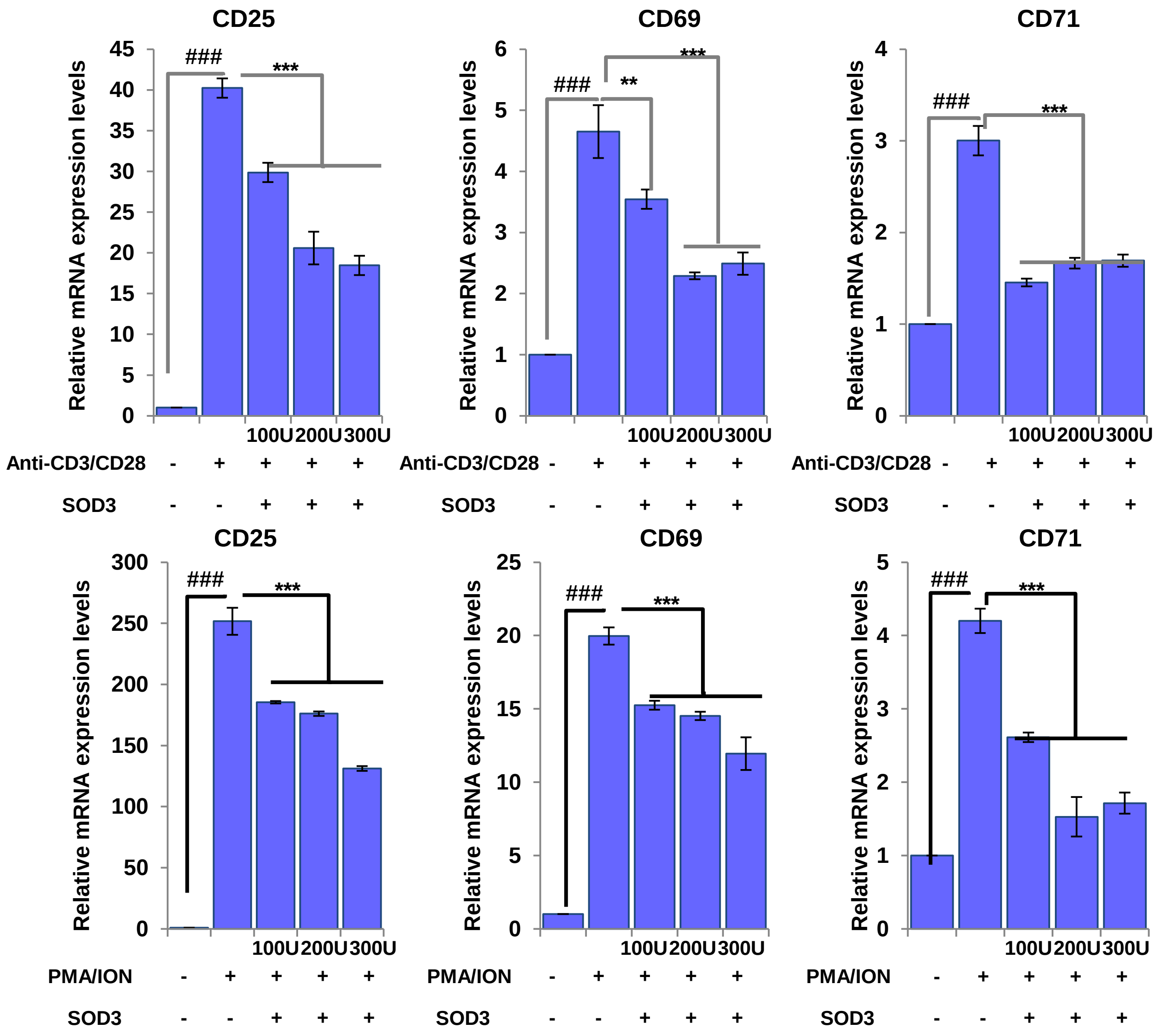

Supplementary Figure S3: Effects of SOD3 and DETCA on T cell activation isolated from SOD3 knock out mice. $CD4^{+}$T cells were isolated from SOD3 knock out and wild type mice first pre-treated with 200 U/mL of SOD3 and DETCA (10 μM) for 1 hour and then treated with (a) anti-CD3/CD28 (3 μg/mL) or (b) PMA (100 ng/mL) and Ionomycin (300 ng/ml) for 24 hours. Expression levels of surface proteins and IL-2 productions were determined by qRT-PCR. All experiments were performed in triplicate. Data are expressed as mean ± standard deviation. $^{\#}p<0.05$, $^{\#\#}p<0.01$, $^{\#\#\#}p<0.001$ (Control group vs. anti-CD3/CD28 or PMA/ION-treated group); $^{*}p<0.05$, $^{**}p<0.01$, $^{***}p<0.001$ (anti-CD3/CD28 or PMA/ION-treated group vs. SOD3 and anti-CD3/CD28 or PMA/ION-treated group); $^{@}p<0.05$, $^{@@}p<0.01$, $^{@@@}p<0.001$ (wild-type vs SOD3 Knock out).

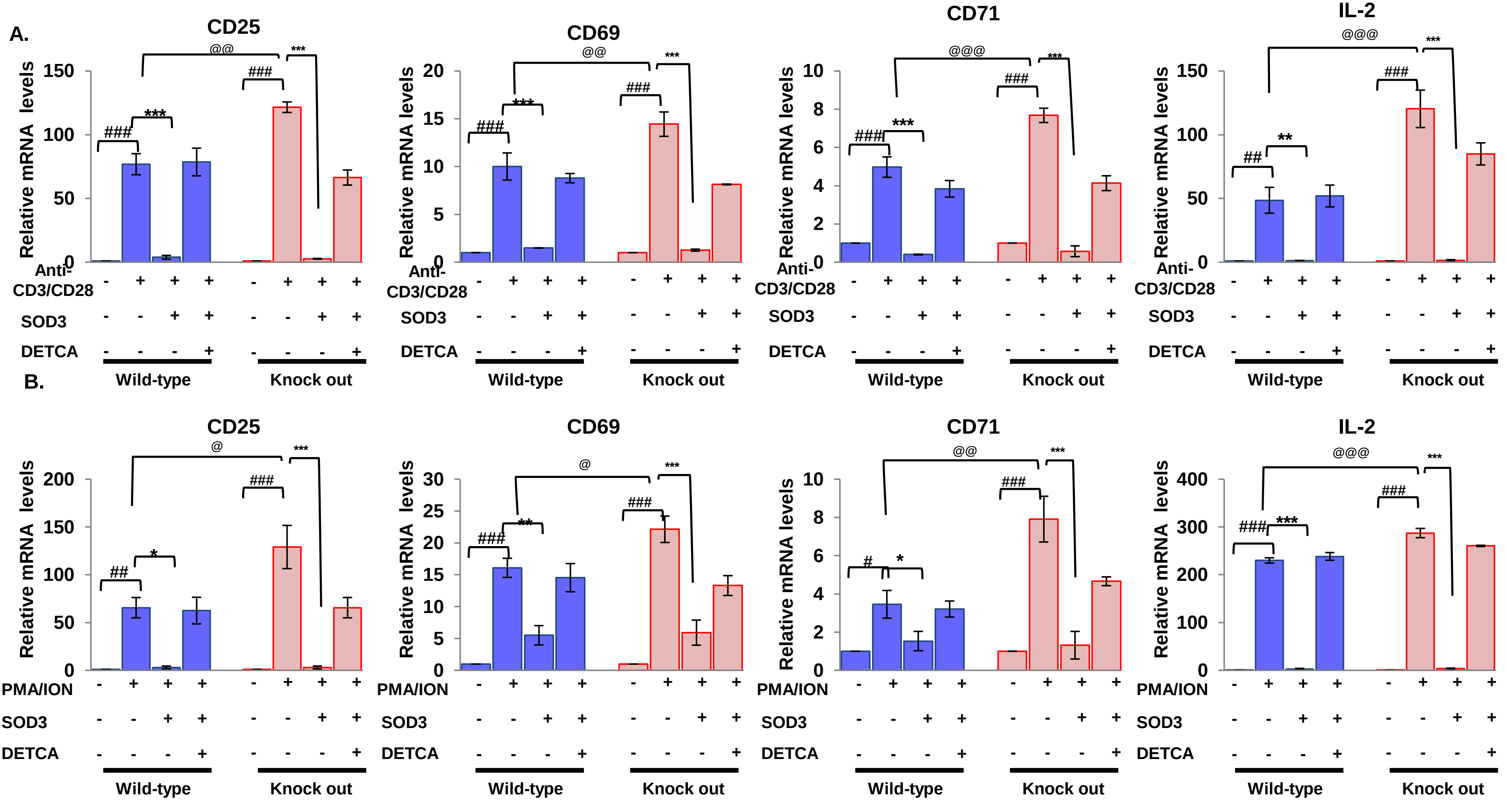

Supplementary Figure S4: SOD3 suppressed the ROS production in T cells. (A) Superoxide anion production in T cells were determined by staining with DHE (10 μM) and measured by fluorimeter at excitation of 500 nm and emission of 600 nm. (B) H_2O_2 production in T cells were detected with DCFDA (10 μM) and measured with fluorimeter at excitation of 485nm and emission of 525nm. All experiments were performed in triplicate. Data are expressed as mean ± standard deviation. $^{\#}p<0.05$, $^{\#\#}p<0.01$, $^{\#\#\#}p<0.001$ (anti-CD3/CD28 vs. SOD3 and anti-CD3/CD28); $^{*}p<0.05$, $^{**}p<0.01$, $^{***}p<0.001$ (Th2vs. SOD3 and Th2); $^{@}p<0.05$, $^{@@}p<0.01$, $^{@@@}p<0.001$ (Th17 vs. SOD3 and Th17).

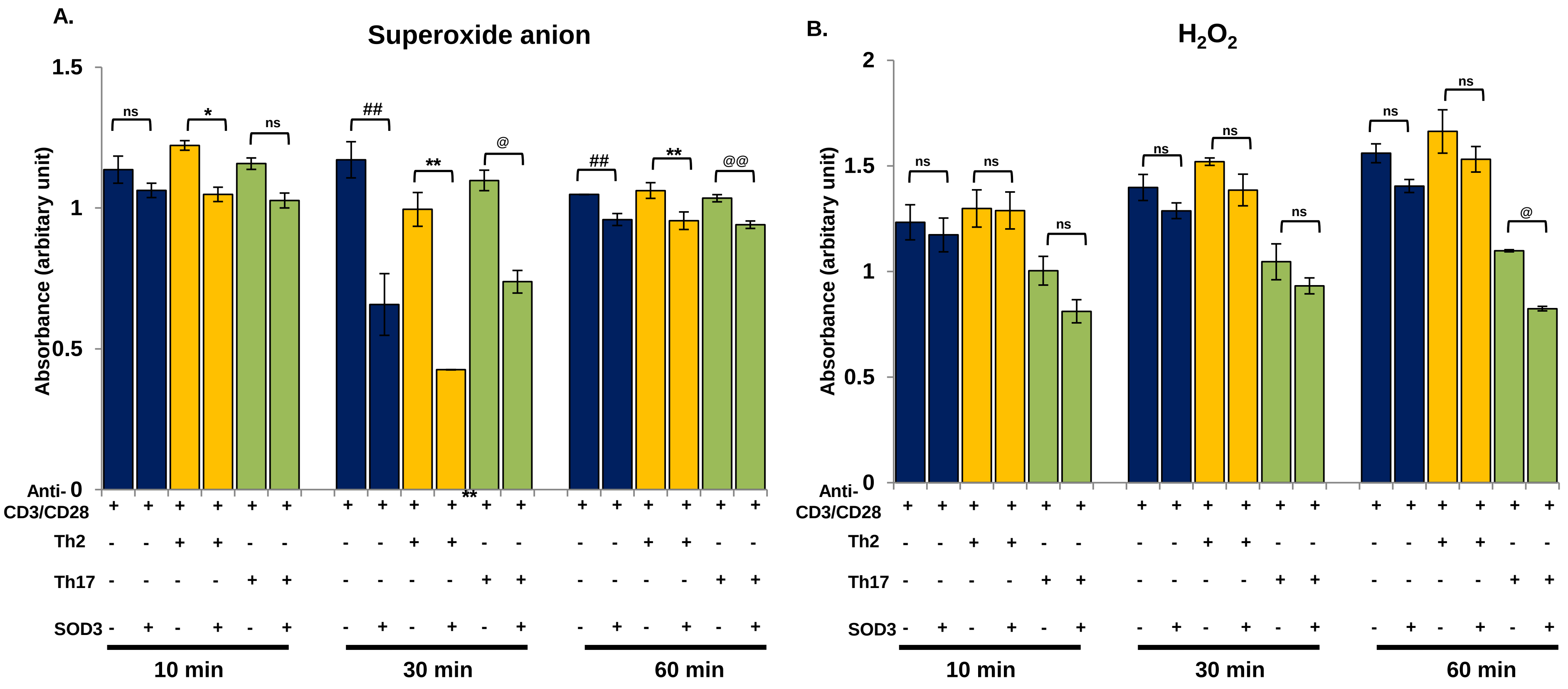

Supplementary Figure S5: protein band intensities of western blot. ImageJ (version 1.46r) software was used to determine the band intensities of western blot data from (A) Figure 4A, (B) Figure 4B, (C) Figure 4C, (D) Figure 4D, (E) Figure 5C. All experiments were performed in triplicate. Data are expressed as mean ± standard deviation. $^{\#}p<0.05$, $^{\#\#}p<0.01$, $^{\#\#\#}p<0.001$ (anti-CD3/CD28 vs. SOD3 and anti-CD3/CD28); $^{*}p<0.05$, $^{**}p<0.01$, $^{***}p<0.001$ (Th2vs. SOD3 and Th2); $^{@}p<0.05$, $^{@@}p<0.01$, $^{@@@}p<0.001$ (Th17 vs. SOD3 and Th17).

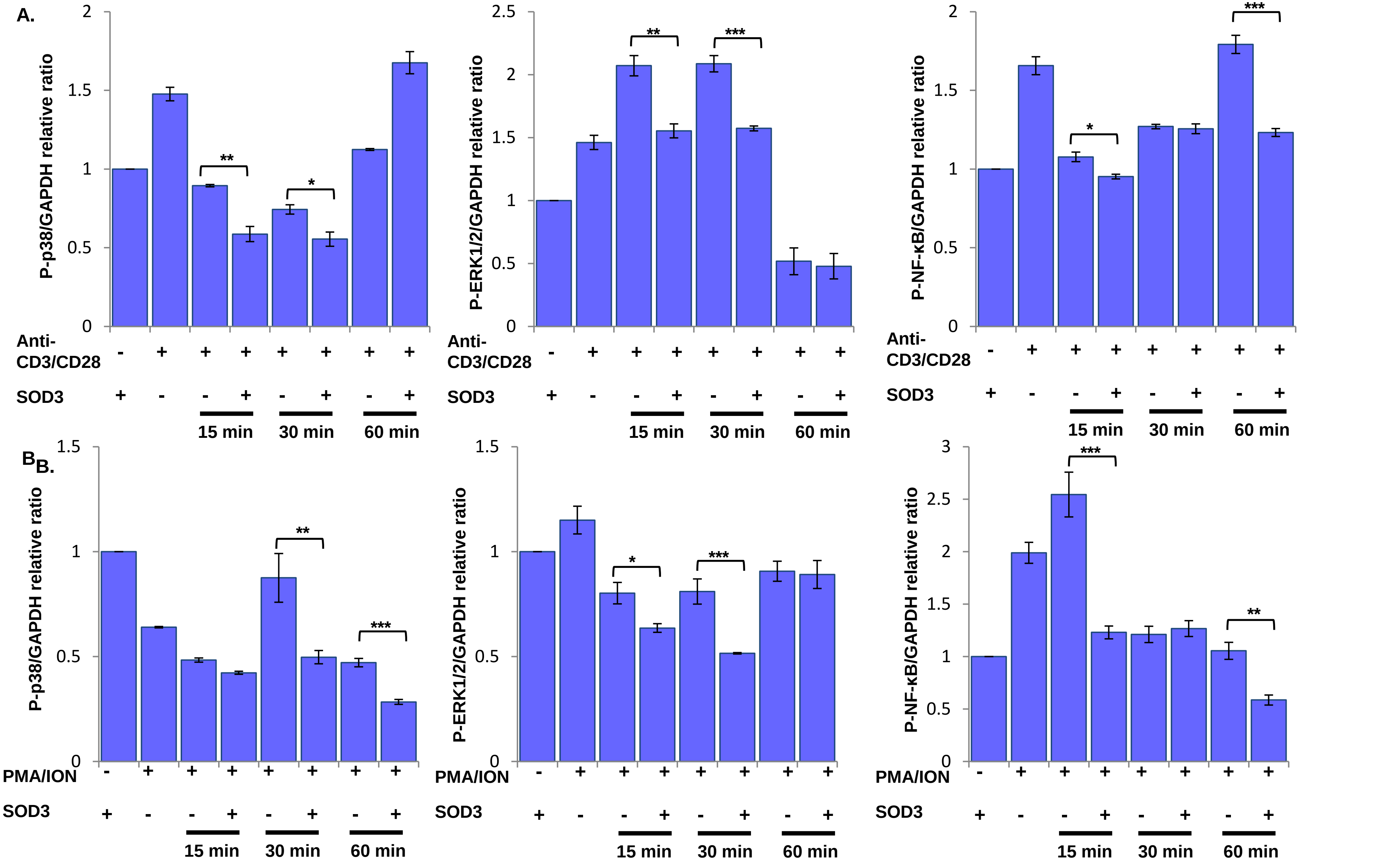

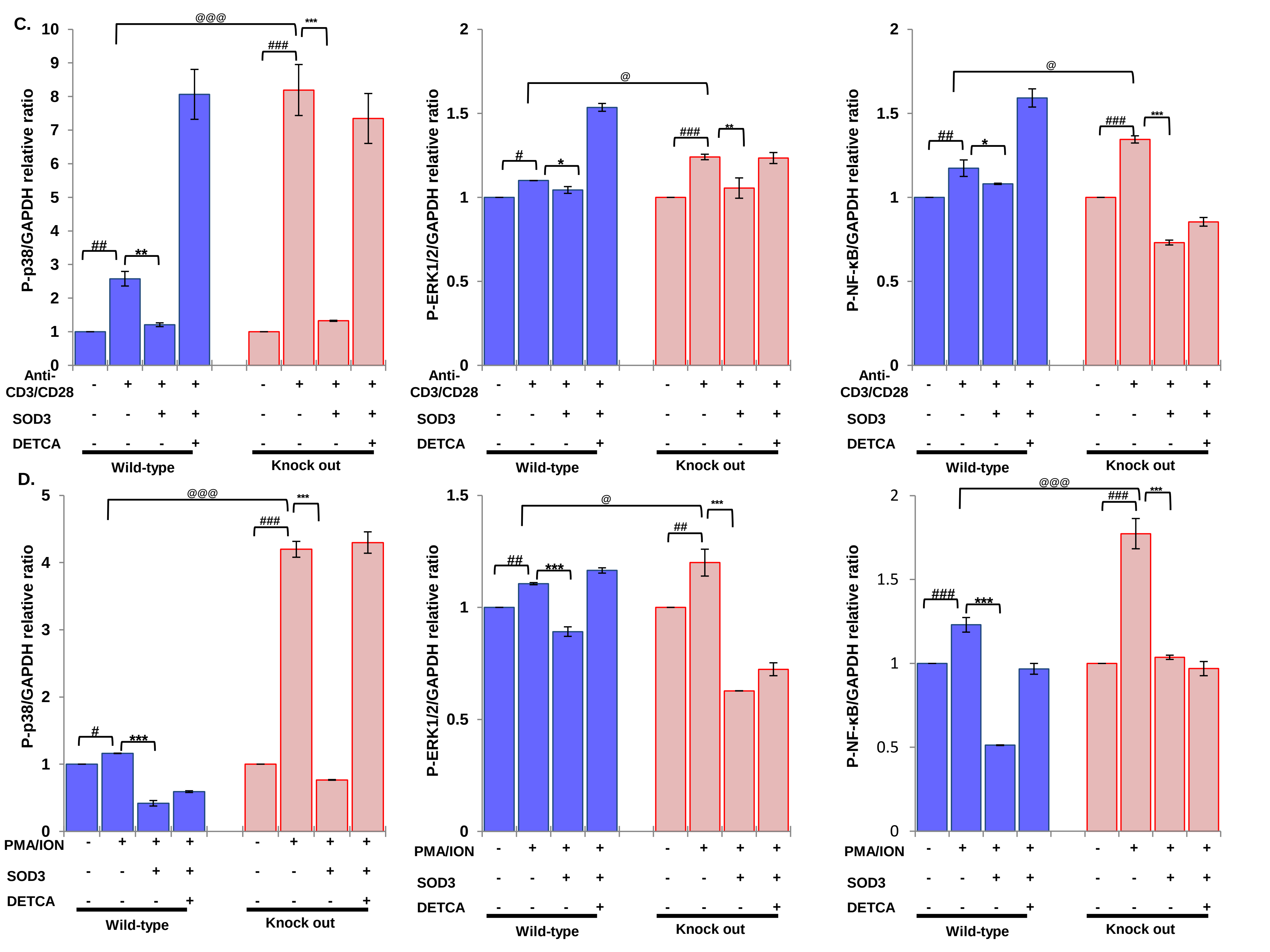
C.
D.
P-p38/GAPDH relative ratio
P-ERK1/2/GAPDH relative ratio
P-NF-κB/GAPDH relative ratio
Anti-CD3/CD28
PMA/ION
SOD3
DETCA
Wild-type
Knock out
@@@
@
###
##
#
***
**
*

E.

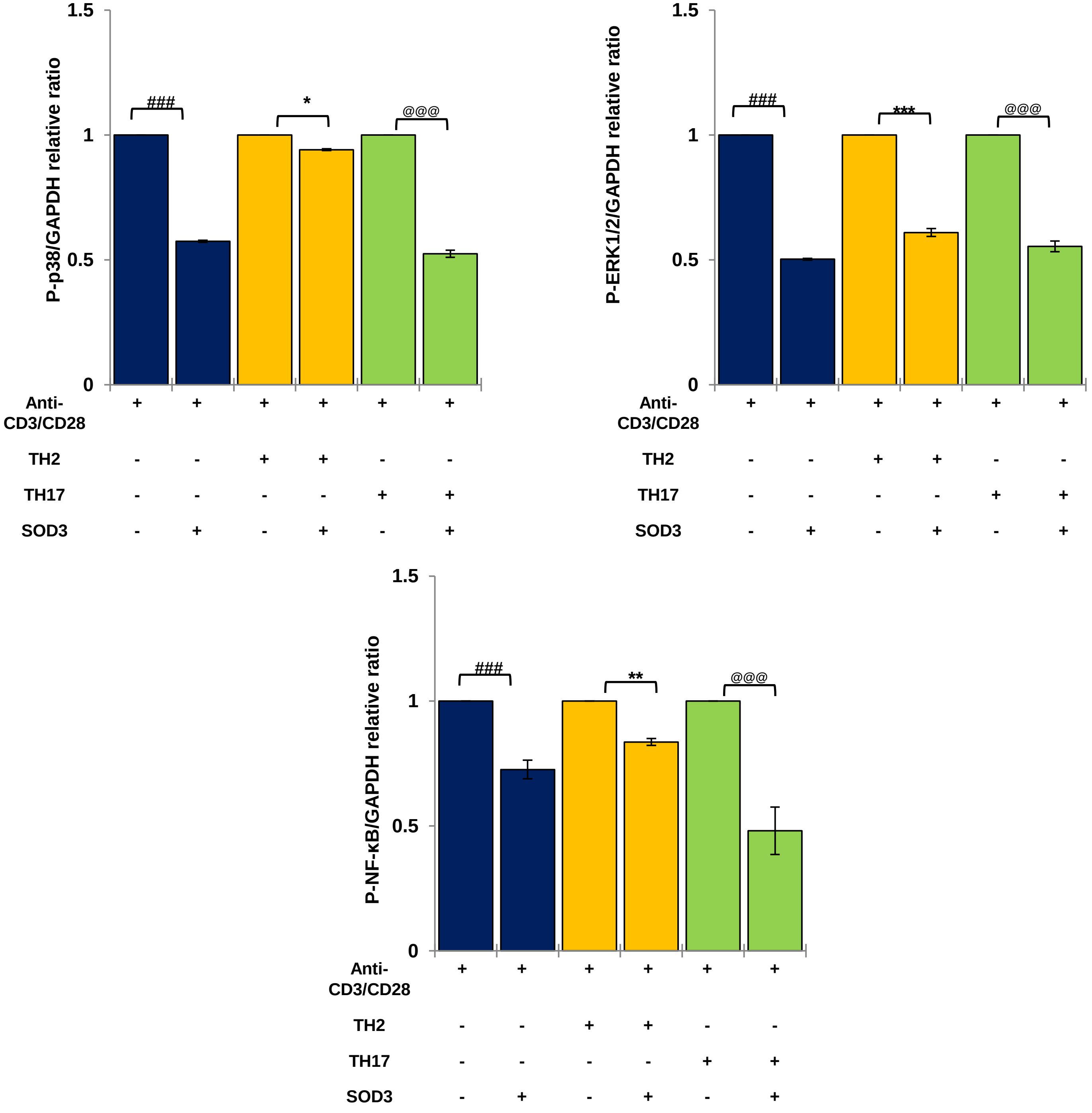
P-p38/GAPDH relative ratio
P-ERK1/2/GAPDH relative ratio
P-NF-κB/GAPDH relative ratio
1.5
1
0.5
0
###
*
***
**
@@@
Anti-CD3/CD28
TH2
TH17
SOD3
