# Supplementary material for: Superoxide Dismutase 3 Controls the Activation and Differentiation of CD4+T Cells
Source: Front Immunol. 2021 Feb 25;12:628117. doi: 10.3389/fimmu.2021.628117 (PMC7947887; doi:10.3389/fimmu.2021.628117)
Supplement: Supplementary file 2 [file Table_1.docx]

**Supplementary Table 1: Primer sequences for quantitative Real-time PCR of mRNA**

| **Primer, Mouse** | **Forward sequences** | **Reverse sequences** |
| --- | --- | --- |
| CD25 | GCGTTGCTTAGGAAACTCCTGG | GCATAGACTGTGTTGGCTTCTGC |
| CD69 | GGGCTGTGTTAATAGTGGTCCTC | CTTGCAGGTAGCAACATGGTGG |
| CD71 | GAAGTCCAGTGTGGGAACAGGT | CAACCACTCAGTGGCACCAACA |
| IL-2 | GAAACTCCCCAGGATGCTCA | CGCAGAGGTCCAAGTTCATCT |
| IL-4 | ATCATCGGCATTTTGAACGAGGTC | ACCTTGGAAGCCCTACAGACGA |
| IL-5 | GATGAGGCTTCCTGTCCCTACT | TGACAGGTTTTGGAATAGCATTTCC |
| IL-6 | TACCACTTCACAAGTCGGAGGC | CTGCAAGTGCATCATCGTTGTTC |
| IL-10 | CGGGAAGACAATAACTGCACCC | CGGTTAGCAGTATGTTGTCCAGC |
| IL-13 | AACGGCAGCATGGTATGGAGTG | TGGGTCCTGTAGATGGCATTGC |
| IFN-γ | CAGCAACAGCAAGGCGAAAAAGG | TTTCCGCTTCCTGAGGCTGGAT |
| TNF-α | GGTGCCTATGTCTCAGCCTCTT | GCCATAGAACTGATGAGAGGGAG |
| GAPDH | CATCACTGCCACCCAGAAGACTG | ATGCCAGTGAGCTTCCCGTTCAG |
